# Turning Community Elder Care into a Profession: Insights from Trainees, Developers, Employers and Supervisors

**DOI:** 10.3390/ijerph17165867

**Published:** 2020-08-13

**Authors:** Aya Ben-Harush, Liat Ayalon, Shiri Shinan-Altman

**Affiliations:** 1 Social Work Department, Faculty of Social & Community Sciences, Ruppin Academic Center, Emek Hefer 4025000, Israel; 2 Preschool Education Track, The David Yellin Academic College of Education, Jerusalem 91035, Israel; 3 School of Social Work, Bar-Ilan University, Ramat Gan 5290002, Israel; liat.ayalon@biu.ac.il (L.A.); shiri.altman@biu.ac.il (S.S.-A.)

**Keywords:** care workers, professionalism, preparation

## Abstract

This study explores the process of turning elder care into a profession, by giving a voice to different professionals who took part in developing and implementing a new Israeli training program for community care workers. The program attempts to offer a response to the shortage of paid long-term carers for older adults by turning community elder care into a profession. Interviews with graduates, trainees who dropped out of the program, developers, employers and supervisors from three regions of the training program were conducted. Analysis explored attempts to transition community care from an occupation to a profession. The community care worker’s role and its uniqueness in comparison to the traditional paid long-term care worker are discussed. The difficulties that stem from the ambiguity of the definition of this new occupation are described.

## 1. Introduction

Most elder care workers are untrained and underpaid [1]. The lack of benefits, such as health insurance and retirement plans further exacerbates paid long-term carers’ low economic position [2]. Hence, recruiting and maintaining stable and sufficient paid long-term carers has become a critical long-term concern worldwide [3]. Scholars pointed out the consequences of this shortage, including increased workloads faced by paid carers and deterioration in the quality of care provided to older adults [4]. As paid elder care is stigmatized [5] and undesired in many countries, worldwide, it is possible that turning elder care into a profession will result in improved status and potentially make it more desirable [6].

In order to shed light on this social problem and consider possible ways to cope with it, there is a need to use a broader context of caring professions, such as nursing, remedial therapies and social work for illustrative purposes. This can allow us to learn from their experiences in establishing their disciplines as a profession. Establishing the paid care of older adults as a profession can potentially attract committed and skilled personnel and will diminish the current stigma towards this occupation.

## 2. Paid Elder Care

Providing care to older adults can be a difficult physical and mental task [7]. Care work often is considered to be a low-status job conducted mainly by women from low socio-economic status [8]. Despite growth in the number of paid long-term elder carers, paid long-term care providers are not clearly defined as belonging to a specific profession [9].

Role ambiguity occurs when the specifications for expected roles are incomplete [10]. Thus, when a paid care worker experiences role ambiguity, he or she may not know what should be accomplished. In order to develop the professional workforce, knowledge of its essence is required.

A crucial way of handling the shortage in paid home care workers can be found in the training of workers and the transformation of long-term care into a profession. Support for this notion may be found in a review of the long-term care workforce [11]. This review offered policymakers worldwide two strategies: (a) improving direct care workers’ job conditions and (b) exploring and evaluating the creation of new pools of supply for the growing demand of this workforce.

In order to view paid long-term care as a profession, there is a need to first define what makes an occupation a profession. Similar to individuals who are competing with each other in the class system, occupations, too, are engaged in the same competition and may move up or down in prestige or income [12].

Therefore, we present the definition of the term profession as suggested by Cruess, Johnston and Cruess [13]: “An occupation whose core element is work based upon the mastery of a complex body of knowledge and skills. It is a vocation in which knowledge of some department of science or learning or the practice of an art founded upon it is used in the service of others. Its members are governed by codes of ethics and profess a commitment to competence, integrity and morality, altruism, and the promotion of the public good within their domain”.

In contrast, the definition of an occupation is narrower, the use of the term occupation is relatively restricted to mean a gainful employment or paid work [14].

In this paper, we examine the development and implementation of a new Israeli training program for community care workers of older adults. The term community care work was selected in order to emphasize the aims of diminishing older adults’ loneliness and integrating them into society. A major goal of the program was turning paid long-term elder care work into a profession. As such, it includes theoretical contents such as nursing courses as well as practical material concerning long-term care settings [15].

The role of the community care worker is to create optimal communication with the client while paying close attention to his/her competencies and limitations. The community care worker is expected to communicate with the client and to plan activities that aim to improve the client’s well-being. In comparison to the role of the community care worker, the role of the traditional paid long-term care worker is more concrete and limited to physical care: “Paid long-term carers assist people who have functional limitations due to advanced age, disability, illness or any other reason, and need assistance in order to perform basic activities” [16].

In Israel, where the present study takes place, there are local traditional elder paid care workers. Their work is characterized by a low status, low salaries, inferior employment conditions, and a lack of promotion opportunities [17]. These workers are mainly middle-aged and older women who immigrated from the former Soviet Union. They work on average 23 h per week and are paid at the minimum level of pay and their salary is paid through the National Insurance Institute of Israel. However, the majority of elder paid care workers are employed through home care agencies, which are responsible for the placement of the carers in the homes of older adults in need [15].

Our claim is that elder long-term care in Israel is currently in limbo between being an occupation and a profession. Through the analysis of qualitative interviews with various stakeholders who participated in the training program, we discuss the circumstances which hinder the ability of the occupation to turn into a profession as well as the components that advance the occupation’s potential for turning into a profession.

## 3. Methods

### 3.1. Sample

The present study is based on data from the evaluation process of a training program for community carers. In the current study, only interviews that addressed aspects of professionalism regarding long-term care were selected in advance. Thus, the sample in this study consisted of 5 groups: (a) graduates of the training program (*N* = 3); (b) trainees who dropped out of the program (*N* = 3); (c) supervisors (*N* = 8); (d) developers (*N* = 1) employers (*N* = 4) (Table 1).

### 3.2. Procedure

#### Data Collection

Given that there were unequal numbers of individuals by each category (trainees and trainers), we used different approaches to data collection. A research assistant conducted focus groups with the trainees (without the presence of staff members). All trainees signed a consent form. Phone interviews were conducted with trainees who dropped out of the program. Structured face-to-face interviews were conducted with staff members and employers. The protocol study was approved by the Ethics Committee of the PI’s university.

Interview guides with developers and the operating team addressed their vision for the program, assets and limitations of the program, as well as potential ways to improve it. Participants were asked about their decision to join the program, the perceived benefits from it, their interest in working with older adults, strengths and weaknesses of the training program and future aspirations.

Analysis was conducted by the first author and was critically appraised by the other two authors who were involved in data collection and analysis for other purposes. First, all data were read carefully and specific data that were more relevant to the transition from an occupation to a profession were chosen. Data were then organized according to central topics based on an “open coding” approach which is sensitive to content and is not affected by predetermined conceptions [18]. For example, integral components of the training program mentioned by informants were named and grouped according to whether they advanced or hindered the development of long-term care as a profession. Finally, the broad list of topics was grouped into three main themes that form the “story line” [19]. Anonymity was kept in the presentation of the findings.

## 4. Findings

Three main themes emerged from the data. The first theme identified the need to establish the role of the community care worker as a profession; this theme comprised two sub-themes: a lack of differentiation between the work of a paid care worker and the work of a community care worker; and the need for marketing the role of the community care worker.

The second theme addressed the central components inherited in the work of the community care worker that turn this occupation into a profession. This theme comprised two sub-themes: distinguished knowledge that draws from different disciplines and fields that are relevant to older adults’ life; and a deep commitment to the well-being of older adults in different areas of life.

The third theme focused on the significance of the training program as a central platform to establish community elder care as a profession. This theme is comprised of three sub-themes: locating and selecting high quality and motivated workers; socializing workers to the role of professional workers; and establishing ongoing connections with potential employers.

### 4.1. Theme 1: Identifying the Need for Establishing the Occupation of Community Care for Older Adults as a Profession

Observing the current status of the occupation of community care for older adults stresses the need to establish the occupation as a profession. The need for such a transformation is derived from two main challenges that characterize the lack of professionalism inherited in the care of older adults at the present time: first, the lack of differentiation between the work of a traditional paid care worker and that of a community care worker; and second, the need for marketing the role of the community care worker.

Today, the occupation called community care is completely unregulated and does not have even minimal employment requirements as it is not considered a profession. Establishing care work as a profession is the only way to create a significant change in the field. A developer of the training program stated:

“The two fields in Israel that are not regulated and are not standardized at all, are caring for toddlers…and caring for older adults. Anyone can offer care without any authorization or supervision, there are no terms for acceptance, required knowledge or even work experience. Caring for older adults…requires training…requires a definition”.

On the vagueness and the labeling surrounding the occupation, one can learn from the following sub-themes:

a. *“What activity are you talking about? Go give the man a shower!”*: A lack of differentiation between the work of a traditional paid care worker and a community care worker.

The occupation of community care is not only an alternative but a significant expansion of the traditional paid care worker’s role. Nevertheless, neither the definition of the community care occupation, nor its distinction from traditional paid care work was clear to the participants in the training program or to potential employers. A reinforcement of this claim can be found in the experience shared by a community care worker:

“We had one community care worker… he was frustrated. You see all the time his strong will to assist and care, but employers stepped on him at work. They used to laugh at him, when he wanted to administer an activity, they told him: ‘You think there is time for these things? Go give the man a shower!’ … How can we use what we have learnt if they don’t let us?”

The ambiguous definition of the community care role also was conveyed by employers. An example is found in the words of an employer in a home care agency:

“One of them started by doing cleaning work for the elders, and later on, she succeeded to do work beyond that. They did not have a chance to bring the contents they learned in the course … I am not sure that community care workers can become integrated as home care workers in the community. People that live in their homes and are not disabled do not need a worker who has gone through a special course.”

One of the supervisors claimed that employment places do not fully understand the unique qualifications of community care workers:

“Because it is such a young program, they don’t know how to use it … People today look for the paid care worker, that will only give a shower and that’s it. The true expansion of the role does not exist yet. That’s why I don’t know what will happen with them when they join the workforce.”

In the light of these quotes that unfold the difficulties embedded in implementing the occupation termed “community care” in the professional field, we claim that creating a clear and defined profession will lead to an acknowledgement that community caregiving is a separate profession from the narrower occupation of paid elder care.

b. *“What are you doing with these elders?”:* the need to market community care as a profession.

Examples of the negative labels that accompany working with older adults can be found in the reports of both training staff and community care workers:

“Someone wants to tell a friend about her work, and then the friend mocks her: ‘What are you doing with these elders? What does it have to do with you?’”

This aspect of negative social reaction towards people who are working with older adults is also expressed in the description of another supervisor:

“If you ask them if their friends know what they learn, their answer is ‘no’. I suppose it is embarrassing to work with older adults when you are 20 years old … being a waitress is considered much better than caring for an older woman”.

The stigma and the low status of the occupation cross different sectors in Israeli society is expressed in the following description given by a Bedouin community care worker:

“My friends … Bedouins and Arabs … do not know what this job is … they laughed at me when they realized that I work with older adults …”

One of the pedagogic coordinators believes that the way to create a more prestigious reputation for the community care worker is strongly linked with creating a social change:

“It is a story of meaning and the will to meet people … I also experience it in the recruitment meetings we conduct … you hear of many courses offered to young people, these courses are practical and important … and when you tell them, ‘I will offer you all these things and also a job with a purpose which is connected to our society and offers a meaningful social connection’. Then you see their eyes open”.

A supervisor in the program, also discusses the need for changing the way the profession is marketed:

“Public visibility. People should know that this thing exists. For example, in one nursing home they chose a very simple act that truly changed the reputation of the profession, they made a name tag: ‘Luna—a community care worker’… It gave the trainees a feeling of pride … clearly recognizing your importance, giving you a place”.

In summary, there is a need for a different and more prestigious marketing strategy of the occupation that will stress its goals, contribution to society and the deep sense of belonging it offers in order to turn it into a profession. As such, the new marketing strategy should assist in diminishing the negative labeling that accompanies working with older adults.

### 4.2. Theme 2: What Makes Community Care a Profession?

The combination between the unique knowledge base that stems from several professional fields that are relevant to the care of older adults, together with the community care workers’ deep commitment to the well-being of their clients in all spheres of life, makes this occupation a profession:

a. *“Seeing the older person as a whole”:* unique knowledge that stems from diverse disciplines that are relevant to older adults’ care.

A profession is built on broad and rich contents that integrate all aspects of the older adult’s life and physical and social environments. The stories of community care workers point to the way all these diverse aspects were brought up during the training program:

“The program was very rich. We had a nurse who taught us all about the physical aspects of care. We had a social worker … she had taught us ethics—how to act in front of a family member … We spent a whole day in a clinic that checks older adults … Occupation, art, music … we experienced being in the older person’s shoes—how do older adults feel when their senses are weak …”

Employers who agreed to hire graduates of the training program were not familiar with the occupation ‘community care worker’, yet, acknowledged the ways in which carers apply their unique knowledge. A manager of a day center elaborates regarding the process of hiring a community care worker:

“I did not know what I was getting into … she does exactly the job that I needed someone to do. She is very oriented to care. The workers in day centers are very service-oriented at a very technical level … she fills the very important function of caring: to stimulate older adults, talk to them, conduct with them different sorts of activities”.

The unique skills and expertise of community care workers are stressed in another employer’s description:

“This community care worker has more motivation and a great will to learn…she works with groups of people with cognitive deterioration, she is the only worker who leads activities in a small group, and it is amazing!”

b. *“To surround all the subject s… also to understand his soul”:* a deep commitment to the client’s well-being in all areas of life.

The community care workers’ specific skills enable them to develop awareness to older adults’ overall well-being, and to perceive themselves as committed to offer assistance in different aspects. The reports of a community care worker draw a picture of dedicated and dignifying care, tailored to the specific needs they recognized in their clients:

“We learned how to motivate the older adult to want to get up in the morning and feel that he/she is alive. Not to look at him/her as if … ’you are kind of a machine—shower you, feed you, give you peels, put you in bed—and goodbye’. It’s a lot more than that”.

Furthermore, in parallel with the transformation that the client is going through as a result of the contact with the community care worker, the worker herself is experiencing a transformation. Ashraf shares his perceptions regarding the essence of the community care worker’s role and elaborates regarding the professional development process he had experienced as a community care worker:

“Instead of bringing a person that doesn’t understand the language and will only sit with him—it’s not enough. We need to sit with the client to understand his needs … the community carer experiences a transformation like every person that cares for other people does … Before [the training], I used to shower, feed, but had no other tools, didn’t understand law. [Now] I have tools”.

In the eyes of another community care worker, her deep commitment to her client is strongly linked to knowing his past; a process that enables her to perceive him as a whole person. These dimensions of the job, that stress the holistic perception of the client and the workers’ ability to cherish the individual life history of every patient are what characterize community caregiving as a profession.

Bat-Sheva elaborates:

“The ability to approach an older adult, talk to him, not to see him and ignore, not to realize that he does not want to speak. To smile, approach him, touch. A community carer should see the entire person … To try to stimulate him to talk, to ask him about his strengths …”

### 4.3. Theme 3: The Training Program as a Platform for Turning the Traditional Occupation of Paid Elder Care into a Profession of Community Care

A high-quality training program is considered essential for improving the current situation in the field of long-term care for older adults. This notion has been interwoven in the stories of trainees, staff and employers. From their interviews, it is apparent that the training program has several roles: locating and selecting high quality and motivated workers; developing socialization to the role of the community carer; and establishing frequent contacts with potential employers.

a. Locating and selecting high-quality and motivated workers.

Although several eligibility requirements (e.g., 12 years of schooling, an interview by the admission committee) are stated, these requirements are not enforced and trainees are accepted regardless of whether they meet these requirements. Furthermore, some of the trainees in the program are coping with cognitive and mental disabilities, and their ability to care for another person is questionable.

“There were in the program some guys who not everything was all right with their heads … I don’t know how they were accepted to such a course, how can you count on such a person to take care of someone else? A person that is going to support another person should himself be all right” (A trainee who left the training program).

Acknowledging community caregiving as a profession will allow for setting a higher standard regarding the qualifications of potential employees, will deepen the understanding of employment places regarding the complexity of this occupation, and may advance the process of finding the resources for creating the required positions of community care workers in the field.

As can be seen, some of the employers have not yet understood the essence of the community care worker’s role. Daphna is the head of a day-care center that collaborated with the training program:

“It’s a course of community care workers. They conducted meetings with my older adults … My older adults used to come to them once a week”.

One can assume that the fact that Daphna did not attend these meetings herself has contributed to her difficulties to understand the essence of the community care worker’s role. In an answer to a question regarding the differences between a community carer and a paid long-term carer, she answers:

“I really don’t know. The paid care worker works in the community, it’s like a community worker, now that I think of it. Maybe the difference is that it’s not an individual work with a client?”

Both participants and staff of the training program stress the importance of locating and selecting high-quality and motivated workers. The tremendous investment in people who do not meet the minimum required conditions for such a position and leave the program before starting to work in the field has been a waste of resources and money.

b. Socializing to the role of a professional worker.

The training program should involve the trainees in a socialization process based on the goal of turning them into professional workers who are able to face the basic requirements of the workforce, such as arrival to the workplace on time and being committed to both client and employer. The relevance of this issue unfolds in an interview with a social worker in a nursing home:

“Some of them, you have to teach and direct them to take responsibility … these things need refreshing, reminding them not only the issue of commitment to work but also to discuss the goals, why she is here, and remind her that she needs to step up and help”.

An interview with a trainee who quit the program sheds light on the complexity of the socialization process. This interview demonstrates the lack of understanding of some trainees regarding the importance of responsibility, commitment and professionalism:

“If we were late by five minutes, they wouldn’t let us in … I thought the course would be interesting, a temporary period … that I would see something else. But I really didn’t enjoy it! We saw a movie … they explained to us—it was very boring! If I knew how it was going to be, I wouldn’t have entered the program to begin with”.

c. Establishing frequent contacts with potential employers.

As part of the training process, the program should build and maintain a connection with potential workplaces and refer the trainees to those places that need workers. On the one hand, the program must make sure that the different workplaces employ the trainees as community care workers and not in the traditional role of paid long-term care workers. On the other hand, the training process should reflect and offer opportunities to discuss the difficulties embedded in assimilating the new profession in the long-term care field.

The program developers point out that some of the centers that operated the program faced challenges regarding the connections they tried to establish with potential workplaces:

“The major challenge is the work placement. We started working with a nursing home. They don’t have any commitment to the program. So we … need to reach every place we find suitable and convince them to accept the trainees. But they are not really part of the program. They have their daily routine and their own ways of training workers” (A developer).

In this context, another developer discusses whether the training genuinely succeeded in preparing the workers to deal with the workplaces’ demands:

“Due to the fact that this role is not simple at all … we thought that they (workers) will perform the different aspects of the job step by step … but we found out that it is more complex than we thought (A developer).

The importance of establishing firm connections with potential employers during the training program is pointed out also by employers. Jonathan, the manager of a day center, describes the process that has led him to hire a community care worker:

“I met the coordinator of the program, and my director believed that we should embrace the program … The added value, regarding the contents administered as part of the program … they seem to me good contents. I wasn’t involved in the program. I know whom I hired—it’s something that is more personal. She works for us at the moment”.

## 5. Discussion

This study explored a new training program for community care workers of older adults through the lens of the term ‘profession.’ The importance of this paper lies in the fact that it attempts to identify a practical solution to the shortage of care workers, by turning community elder care into a profession [20].

Our claim, that community caregiving should be established as a profession, is built upon two main aspects facilitated through the program: the workers’ broad body of knowledge, on the one hand, and the workers’ deep commitment to their clients, on the other hand. The training program was found to be a crucial platform for turning community care into a profession, although it was not entirely successful.

The first theme describes the complexity of the role of the community care worker as a unique and a much broader profession than that of the paid care worker, who mostly focuses on applying physical care. The complexity, that requires special traits and qualifications from the worker, is one of the main reasons why community care should be acknowledged as a profession. The need for transforming the way we perceive care for older adults is discussed in the professional literature. For example, the ‘Stay Active at Home’ program in the Netherlands was designed to train home care workers to focus on the capabilities of older adults rather than on disease and dependency [21]. The authors stress that the program equipped the workers to master particular skills and learn how to deal with challenging situations. This strengthens our claim that the unique sets of skills the community carer applies in his or her daily work with older adults should be acknowledge and established as a profession.

We claim that the ambiguity surrounding the new occupation and the confusion regarding its definition leads employers to expect community care workers to function as long-term care workers, rather than as community care workers. This in turn, leaves the workers frustrated and resentful. This issue was also discussed in a study of U.K. care home staff [22]. They found that although staff was asked to support the personal identities of residents, carers found themselves unable to incorporate their caring values in their work and were required to conform to a more practical form of care, which addressed mainly the physical aspects.

We point out to the need for more prestigious marketing of the profession of community care workers. Marketing should stress the goals of improving the client’s quality of life and the contribution of the profession to society. This is expected to challenge the generally accepted opinion that associates with elder care mainly technical personal care such as bathing and dressing, which then leads to stigmatization of elder care. In this context, scholars explored the subjective world of home care workers of patients with dementia, and found that their need for recognition was crucial [23]. They stressed the significance of having outsiders understand how important it is for them to be able to achieve an improvement in the client’s well-being, however small it may be.

Two central components that make community care a profession were identified: knowledge and commitment. Participants attributed great importance to the unique and relevant knowledge that they applied in order to improve their client’s well-being in different aspects of life. Furthermore, they stressed their deep commitment to the goal of assisting their client to live a dignified life and to be treated with respect, sensitivity and awareness. The same components were discussed in the study of carers in Sweden [24]. The main finding of the study was carers’ strong commitment to their relationship with older people. Authors claim that this commitment is exhibited in the form of carers’ theoretical and practical knowledge.

The third theme sheds light on the importance of the training program as a platform for establishing community care as a profession. Similar conclusions were offered by a report of the Institute of Medicine [25] that called states to establish basic training requirements and competencies to ensure the development of a high-quality home care workforce. In this context, scholars describe the limited attention and investment in competency-based training for this workforce [26]. The current study offers an observation of the development and implementation of such a program and an analysis of the program’s efforts to create a new profession in the field of long-term care for older adults, namely community care.

## 6. Conclusions

Despite these limitations, our study makes a strong case for further research into establishing community care as a profession. The study’s significance is based on identifying the needed transformation of the occupation of paid elder care into a profession. Future research should longitudinally explore the development and progression of the training program and the ability and will of both trainees and employers in the field to embrace the new profession. Policy makers should define the practical translation of perceiving community caregiving as a profession in measurable terms such as salary, basic requirements and standards for the profession, and the optimal marketing of the profession in order to highlight its positive contribution to both clients and society at large.

### Limitations

Despite its strengths, the present study has several limitations. First, we should acknowledge the issue of social desirability. It is possible that some of the respondents focused on the stronger sides of the program because they believed they should justify their efforts. Second, we acknowledge that the same training program worked differently in various centers that had different instructors and different target populations. This aspect should be examined in further research.

## Figures and Tables

**Table 1:** Participants’ characteristics.

	Role in the Program	Gender	Religion	Age	Marital Status	Years of Education
1	Developer	Female	Jewish	not reported	not reported	not reported
2	Supervisor	Female	Jewish	not reported	not reported	not reported
3	Supervisor	Female	Jewish	not reported	not reported	not reported
4	Supervisor	Female	Jewish	not reported	not reported	not reported
5	Supervisor	Female	Jewish	33	Single	18
6	Supervisor	Female	Jewish	35	Single	18
7	Supervisor	Female	Jewish	35	Single + 1	17
8	Supervisor	Female	Jewish	31	Single	17
9	Supervisor	Female	Jewish	31	Single	16
10	Graduate	Male	Arab	39	Married + 3	12
11	Graduate	Male	Jewish	33	Single	12
12	Graduate	Female	Jewish	64	Married + 4	12
13	Employer	Male	Jewish	38	not reported	15
14	Employer	Female	Jewish	41	not reported	17
15	Employer	Female	Jewish	64	not reported	17
16	Employer	Female	Jewish	37	not reported	15
17	Dropout	Male	Jewish	22	Single	12
18	Dropout	Male	Jewish	20	Single	12
19	Dropout	Female	Jewish	20	Single	12
